# DO WARMED BLANKETS CHANGE PAIN, AGITATION, MOOD, OR ANALGESIC USE AMONG NURSING HOME RESIDENTS?

**DOI:** 10.1093/geroni/igz038.2320

**Published:** 2019-11-08

**Authors:** Christine Kovach

**Affiliations:** Ovation Communities, Milwaukee, Wisconsin, United States

## Abstract

Warmed blankets have not been empirically tested for use in long-term care. The purpose of this study was to describe the use of warmed blankets in a nursing home setting and to determine if use was associated with changes in pain, agitation, mood, or analgesic use. Short-term measures were compared from baseline to post warmed blanket use and longer term differences were compared between those receiving warmed blankets and a randomly selected comparison group. Excluded from eligibility were those using a transdermal drug, with an acute injury, acute inflammatory process, multiple sclerosis, open skin wound, or other condition that could be worsened by superficial heat. Measures included the Revised Faces Pain Scale, PAIN-AD scale, the Brief Agitation Rating Scale, and from the electronic medical record one month measures pain complaints, pain severity, and analgesic use. Long-term measures were taken from the electronic medical record. Of the 141 eligible residents, 24.1% (n = 34) received a warmed blanket over the one month study period. There were statistically significant decreases in both pain level and agitation between baseline, 20 minutes after application, and the subsequent shift assessments (p < .001). There were also long-term changes in the number of pain complaints (p = .040), severity of pain complaints (p = .009), and prn analgesic use (p = .011). There were no statistically significant differences between the treated group and comparison group on any long-term measures. Warmed blankets are a low-cost intervention with a high potential for bringing comfort to nursing home residents.

